# Whole Team, Whole Person High-Involvement Quality Improvement Training for VA Community Living Center Leaders

**DOI:** 10.1093/geroni/igab046.271

**Published:** 2021-12-17

**Authors:** A Lynn Snow, Valerie Clark, Shibei Zhao, Ryann Engle, Corilyn Ott, Princess Nash, Lisa Minor, Christine Hartmann

**Affiliations:** 1 University of Alabama, Tuscaloosa, Alabama, United States; 2 VA Bedford Healthcare System, Bedford, Massachusetts, United States; 3 VA Boston Healthcare Center, CHOIR, VA Boston Healthcare Center, CHOIR, Massachusetts, United States; 4 Birmingham VA Medical Center, Birmingham VA Medical Center, Alabama, United States; 5 Tuscaloosa VA Medical Center, Tuscaloosa, Alabama, United States; 6 Geriatrics and Extended Care, Washington, District of Columbia, United States; 7 VA Bedford Healthcare System, VA Bedford Healthcare System, Massachusetts, United States

## Abstract

Long-term care is a challenging environment for quality improvement due to the high resident acuity, wide variation in resident needs, and wide variation in types and backgrounds of the large staff across three daily shifts. We report results from a learning collaborative undertaken to improve care quality and staff quality improvement skills in the VA CLCs through development of high functioning relationally coordinated teams operating in accord with person-centered care principles. The collaborative included 27 CLCs. Over 9 months leadership teams completed action assignments supported by 5 workshops and regular group coaching calls. Evaluation included fidelity monitoring (attendance, mid- and final progress reports), satisfaction questionnaires, and review of the VA quality measures (CLC Compare). Pre-post participant evaluations revealed a significant increase in positive responses to the question “to what extent do you think applying these new skills/knowledge will improve quality in your CLC?” and positive responses trending toward significance in ratings of abilities to apply new skills. Open-ended survey comments were positive and indicated change in understanding and practice: “utilizing the daily huddle to facilitate real time communication afforded the team a proactive approach to providing care and reducing acute exacerbations. We are able to avert, evaluate as a real time team and make it happen in the now not as a look back.”; “definitely unified front-line staff and CLC leadership.” Some changes were achieved in CLC Compare quality scores (e.g., falls with major injury rate had a 9.6 reduction (average rate = 3.39 pre, 3.07 post)).

